# *Bacillus thuringiensis* Maize Expressing a Fusion Gene *Cry1Ab/Cry1AcZM* Does Not Harm Valued Pollen Feeders

**DOI:** 10.3390/toxins11010008

**Published:** 2018-12-26

**Authors:** Xiaowei Xie, Zhifu Cui, Yanan Wang, Yuanyuan Wang, Fengqin Cao, Jörg Romeis, Yufa Peng, Yunhe Li

**Affiliations:** 1 College of Environment and Plant Protection, Hainan University, Haikou 570228, China; m13126557379@163.com (X.X.); zhifu555@163.com (Z.C.); 2 State Key Laboratory for Biology of Plant Diseases and Insect Pests, Institute of PlantProtection, Chinese Academy of Agricultural Sciences, Beijing 100193, China; wynwynwyn11@163.com (Y.W.); yuanyuanw09@163.com (Y.W.); yfpeng@ippcaas.cn (Y.P.); 3 Agroscope, Research Division Agroecology and Environment, 8046 Zurich, Switzerland; joerg.romeis@agroscope.admin.ch

**Keywords:** Non-target effect, ELISA, *Bt* maize, ladybirds, Bees, Lacewings

## Abstract

The ladybird *Propylea japonica*, adults of the green lacewing *Chrysoperla nipponensis* and the honey bee *Apis mellifera* are common pollen feeders in many crop systems. They could therefore be directly exposed to Cry proteins in *Bacillus thuringiensis* (*Bt*)-transgenic crop fields by ingestion of pollen. They, or closely related species, are therefore often selected as surrogate test species in non-target risk assessment of *Bt* plants. In the current study, we evaluated the potential effects of the ingestion of *Bt* maize pollen containing the Cry1Ab/Cry1Ac fusion protein on various life-table parameters of the three pollen-feeding non-target species in laboratory feeding assays. The results showed that pupation rate and male adult fresh weight of *P. japonica* were significantly increased when fed pollen from *Bt* maize compared to control maize pollen, but other test life-table parameters were not affected. For the other two species, none of the tested life-table parameters (survival, pre-oviposition period, fecundity and adult fresh weight for *C. nipponensis;* survival and mean acinus diameter of hypopharyngeal glands for *A. mellifera*) differed between non-*Bt* and *Bt* maize pollen treatments. ELISA measurements confirmed the stability and uptake of the Cry protein by all three species during the feeding bioassays. In addition, a sensitive insect bioassay confirmed the bioactivity of the Cry1Ab/Cry1Ac protein in the *Bt* maize pollen used. Overall, the results suggested that the three pollen feeders are not sensitive to the Cry1Ab/Cry1Ac protein, and planting of the *Bt* maize variety will pose a negligible risk to *P. japonica*, adult *C. nipponensis* and adult *A. mellifera*.

## 1. Introduction 

Since first commercialized in 1996, genetically engineered (GE) maize varieties that produced insecticidal proteins from *Bacillus thuringiensis* (*Bt*) to control Lepidoptera and/or Coleoptera pests are planted on increasing areas worldwide [1,2]. The large-scale planting of *Bt*-transgenic maize varieties has led to area-wide declines in target pest populations and economic benefits to growers [3,4]. However, any new genetically engineered plant must undergo an environmental risk assessment prior to being approved for commercial cultivation. This includes in particular an assessment of risks to non-target organisms (NTOs) that fulfill important ecological functions [5,6,7,8]. The evidence available today indicates that the currently deployed *Bt* proteins to protect maize from target pest damage cause no direct toxic effects to non-target organisms [9,10,11,12,13,14,15,16].

While China has not grown any GE maize to date, great efforts have been devoted to develop Lepidoptera-resistant lines. *Bt*-transgenic GE maize lines expressing *cry1Ac*, *cry1Ah*, *cry1Ie*, *cry1Ab/cry2Aj*, or *cry1Ah/cry1Ie* have shown to be highly effective against Lepidopteran pests [17,18]. One *Bt* maize line (ZZM030) expressing a fusion *Cry1Ab/Cry1AcZM* gene has recently been developed, and studies assessing its effects on non-target arthropods are currently lacking. 

The assessment of non-target effects focuses on species that have economic or ecological value and that are likely to be exposed to the insecticidal compound produced by the plant [19,20,21]. These include natural enemies such as predators and parasitoids that help control herbivores, pollinators, and species that support nutrient cycling, i.e. detritivores. The ladybird beetle *Propylea japonica* (Thunberg) (Coleoptera: Coccinellidae) is an important predator in agro-ecological systems [22,23]. Both larvae and adults prey on young larvae and eggs of Lepidopterans and other pests [24] and feed on plant pollen (including maize) for its complementary nutrition during plant anthesis [25]. Therefore, the ladybird is exposed to insecticidal proteins produced by *Bt* maize directly by using pollen or indirectly by feeding on herbivores. Similarly, *Chrysoperla nipponensis* (Tjeder) (syn.: *C. sinica*) (Neuroptera: Chrysopidae) also has the potential to be exposed to insecticidal proteins as larvae when feeding on soft-bodied prey, and as adults when consuming pollen [26,27]. Maize pollen appears to be a particularly important and suitable food source for adult green lacewings [26]. The honey bee *Apis mellifera* adult (Hymenoptera: Apidae) is a pollinator of ecological and economic importance that also consumes maize pollen. For this reason, *A. mellifera* is commonly used to assess the non-target effects of *Bt*-transgenic crops [9,20,28,29,30,31]. All three species are thus suitable surrogate test species to support the non-target risk assessment of *Bt* maize that expresses the *cry* genes in the pollen. 

In the current study, we investigated the potential effects of *Bt* maize pollen containing the Cry1Ab/Cry1Ac protein on pollen-consuming beneficial species including the predators *P. japonica* and *C. nipponensis* and adults of the honeybee *A. mellifera* in the laboratory. 

## 2. Result

### 2.1. Effects on Life-Table Parameters

Over 91% of *P. japonica* larvae survived the larval stage, and over 90% of the pupae resulted in adults when fed with maize pollen (Table 1). For two of the recorded parameters, significant differences between the two pollen treatments were detected. First, pupation rate was higher in the *Bt* pollen treatment compared to the non-*Bt* pollen treatment. Second, male fresh weight was higher in beetles that had developed on *Bt* pollen. None of the other parameters (larval survival rate, eclosion rate, larval development time, female fresh weight, pre-oviposition period, and total fecundity) differed between the two pollen treatments.

Over 71% and 90% of adult *C. nipponensis* females and males, respectively, survived on maize pollen for more than 28 days (Table 2). None of the recorded parameters (survival, pre-oviposition period, total fecundity, adult fresh weight) differed between the two pollen treatments.

**Figure 1:**
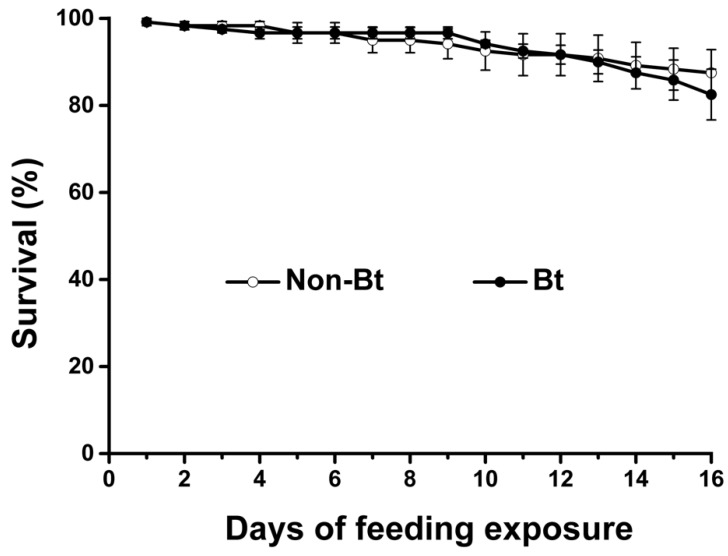
Survival of *Apis mellifera* worker bees fed pollen from *Bacillus thuringiensis (Bt)* maize (ZZM030) or the corresponding non-*Bt* maize (Xiang249). Values are means ± standard error (SE). The experiment was initiated with 60 adults per treatment.

**Figure 2:**
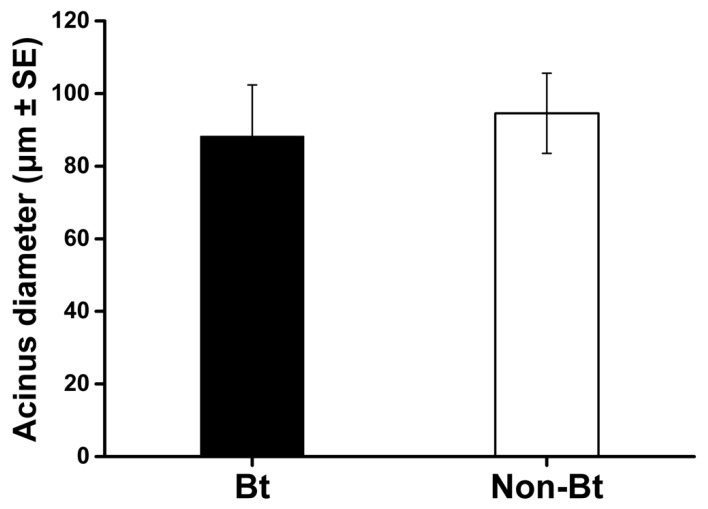
Diameter of hypopharyngeal gland acini of *Apis mellifera* worker bees fed pollen from *Bt* maize (ZZM030) or the corresponding non-*Bt* maize (Xiang249). Values are means ± standard error (SE), *n* = 15.

For *A. mellifera,* over 82% were alive after 16 days of feeding on *Bt* or control maize pollen (Figure 1). The survival rate did not differ significantly between the two pollen treatments (χ^2^ = 0.314; *P* = 0.575). After 10 days of feeding on maize pollen, the mean (±SE) diameter of hypopharyngeal gland acini was 94.6 ± 11.04 μm for non-*Bt* pollen (Xiang249) and 88.2 ± 14.15 μm for *Bt* pollen (ZZM030) (Figure 2). This difference was not significant (Student’s test; *t* = −1.42, df = 30, *P* = 0.52).

### 2.2. Uptake of Cry Protein by Test Species

ELISA analyses confirmed that all test species that had been fed pollen from *Bt* maize contained Cry1Ab/Cry1Ac. No Cry1Ab/Cry1Ac protein was detected in the insects that were fed control pollen. At the end of the feeding experiments, the mean concentration (±SE) of Cry1Ab/Cry1Ac in female and male *P. japonica* was 2.40 ± 0.36 μg/g fresh weight (FW) and 2.18 ± 0.67 μg/g FW, respectively. In the case of *C. nipponensis*, concentrations were 2.08 ± 0.11 μg/g FW and 0.71 ± 0.27 μg/g FW for females and males, respectively. For *A. mellifera*, the mean (±SE) concentrations of Cry1Ab/Cry1Ac were 2.18 ± 0.17 μg/g FW, 1.21 ± 0.32 μg/g FW, and 1.49 ± 0.11 μg/g FW after they had fed *Bt* maize pollen for 5 d, 10 d, and 15 d, respectively. 

### 2.3. Stability of Cry Proteins in Maize Pollen during the Feeding Exposure

After being exposed to the condition of 26 ± 1 °C, 75 ± 5% RH and 16:8 h L:D photoperiod for the feeding experiments with *P. japonica* and *C. nipponensis* for two days, the mean (±SE) concentration of Cry1Ab/Cry1Ac in *Bt* maize pollen was 40.12 ± 3.40 μg/g DW, which did not significantly differ from the concentration in the pollen before being exposed to the insects (42.31 ± 3.60 μg/g DW) (Student’s *t*-test; *t* = 0.44, *df* = 4, *P* = 0.94). In the bee bioassay, ELISA analysis revealed that the original concentration (±SE) of Cry1Ab/Cry1Ac in ZZM030 pollen was 43.10 ± 2.04 μg/g DW. After being exposed to bees for one day, the concentration was 39.58 ± 2.49 μg/g DW, and this change was also not significant (Student’s *t*-test; *t* = 0.85, *df* = 2, *P* = 0.49). 

### 2.4. Bioactivity of Cry1Ab/Cry1Ac in Maize Pollen during the Feeding Exposure

The mean (±SE) fresh weight of *Bt*-sensitive larvae of *C. suppressalis* was significantly reduced when they had fed on an artificial diet containing pollen from *Bt* maize (ZZM030) that was fresh (0.17 ± 0.03 mg) or that had been exposed in a climate chamber at 26 ± 1 °C, 75 ± 5% RH and a 16:8 h L:D photoperiod for two days (0.21 ± 0.03 mg) when compared to larvae fed on diet containing fresh pollen from the non-*Bt* maize (Xiang249) (3.80 ± 0.63 mg) (Student’s t test; *t* = 5.67, *t* = 5.75; both *P* < 0.01). The mean weight of *C. suppressalis* larvae did not differ significantly between fresh and two-day old *Bt* maize pollen (*t* = 0.98; *P* = 0.38).

## 3. Discussion

There is a great variety of arthropods present in agro-ecosystems, but only a small fraction of species can be tested to support the non-target risk assessment of *Bt* plants. Species are thus selected that contribute to important ecological functions, have economic or aesthetic value, are rare or endangered, are likely to be exposed to the insecticidal protein(s) expressed in the crops, and are available and amenable for testing under controlled laboratory conditions [20,25]. In the present study, three non-target species (*P. japonica*, *C. nipponensis*, and *A. mellifera*) were selected because of their economic and ecological value and because they are pollen feeders and will be directly exposed to the Cry protein produced by *Bt* maize ZZM030 in the pollen [32,33,34]. All three species have previously been used as surrogate test species and test systems have been established [11,25,31,35]. 

From the various life-table parameters of *P. japonica* assessed in the feeding bioassay, most were not affected by ingestion of *Bt* maize pollen. This includes survival, pupation rate, developmental time (days from neonate to pupa), adult fresh weight of females, and fecundity. However, the male adult fresh weight and pupation rate were significantly increased by 13.3% and 18.9%, respectively, when ladybirds were fed with Cry1Ab/Cry1Ac-containing maize pollen compared to control pollen. These results are in line with previous studies that showed that *P. japonica* is not adversely affected by Lepidoptera-active Cry proteins including Cry1Ab, Cry1Ac, Cry1F, Cry1Ie, Cry1C, or Cry2A [36,37,38,39]. The observed differences between the *Bt* and the non-*Bt* pollen are likely due to unknown differences in the nutritional composition of the two pollen types as has previously been observed [36,38,40]. Moreover, the observed differences are unlikely to be of relevance under field conditions, because the predator only deploys maize pollen as a supplementary food during a short period of time. In fact, the pollen shedding period of maize usually lasts only for 5–8 days with a maximum of 14 days [41]. Our feeding experiments all lasted for more than 14 days to ensure worst-case exposure conditions. 

In the case of *C. nipponensis*, our results revealed that the ingestion of Cry1Ab/Cry1Ac-containing transgenic maize pollen did not significantly affect any of the adult life-table parameters compared with the corresponding non-*Bt* maize pollen treatment. This is in accordance with the majority of literature showing no adverse effect of Lepidoptera-active Cry proteins including Cry1Ab, Cry1Ac, Cry1C, Cry1F, Cry2Aa, and Cry2Ab on different lacewing species [26,27,35,42,43,44,45]. Two exceptions to this exist. First, feeding assays with larvae of *C. carnea* and purified Cry1Ab had reported putative adverse effects on the predator [46]. These effects, however, could not be verified in numerous follow-up studies [27]. Second, while negative effects of Cry1Ab-containing maize pollen from MON810 on adults of *Chrysoperla plorabunda* (Fitch) were detected in a study by Mason et al. (2008), the authors suggested that this effect was not caused by the Cry protein itself, because the effect was not observed when insects were fed pollen from *Bt* maize event 176, even though it contains significantly higher amounts of Cry1Ab [47].

In the case of *A. mellifera*, consumption of Cry1Ab/Cry1Ac-containing *Bt* maize pollen did not reduce the survival of *A. mellifera* adults during the 16-day bioassay. The test system has been confirmed to be able to detect detrimental effects if present via using potassium arsenate (PA) as a positive control in a previous study [31]. Moreover, no significant effect of ZZM030 maize pollen on the hypopharyngeal gland (HPG) development (as indicated by acinus diameter) of adult bees, which has been confirmed as a sensitive measurement end-point parameter for assessing the potential dietary effects of insecticidal compounds on honeybee adults [31,48], was observed. This result is thus consistent with previous studies in which HPG development was not adversely affected when bees ingested Cry proteins including Cry1Ac (together with CpTI), Cry1Ba, Cry1Ab, Cry1C, Cry2A [28,31,48,49]. 

To clarify the exposure level of *P. japonica*, C. *nipponensis*, and *A. mellifera* in our laboratory feeding experiments, we determined the stability of the Cry1Ab/Cry1Ac protein in the maize pollen diets and the uptake of the Cry protein by the test insects using ELISA measurements. For *P. japonica* and C. *nipponensis*, exposure was relatively stable with 5.2% degradation of Cry1Ab/Cry1Ac protein in *Bt* maize pollen diets during the two-day exposure period. At the end of the experiments, considerable concentrations of Cry protein were detected in adults of both *P. japonica* and *C. nipponensis*. The concentration of Cry1Ab/Cry1Ac protein found in female *C. nipponensis* was about three times higher than in males. This is in line with previous lacewing studies that reported 7–25 times higher Cry protein concentrations in females [32,35]. The reason for the difference between sexes could be that lacewing females consume more pollen as a protein source for reproduction, while males are most likely to consume mainly sugar solutions and only small amounts of pollen to sustain life [26,32,35]. Previously it has been reported that in the case of *P. japonica,* over 76% of the ingested Cry protein was excreted with feces while in the case of lacewings, over 60% was digested during the gut passage of *Bt* maize pollen [26,38]. While it is generally recommended to provide high doses of purified Cry proteins to non-target species to add rigor to the risk assessment studies [11,34], we believe that our studies were conducted under realistic worst-case conditions for two main reasons. First, pollen is not the sole food source of all three species, even during anthesis. Second, in the case of predators, the Cry protein concentration is generally reduced in herbivores when compared to plant material [14]. 

Studies with *A. mellifera* were conducted at a higher temperature (30 °C) compared to the two predators. Not surprisingly, the concentration of the Cry protein in the pollen degraded faster (by 8.2% per day). Similar results were observed in a previous study, where the concentrations of Cry2A and Cry1C declined by 9.9% and 28%, respectively, during a two-day exposure period [31]. Thus, we can expect that the Cry protein concentration in *Bt* maize pollen declines even faster when the pollen is stored in the bee hive at 34 °C and 75–90% RH. The ingestion of the Cry1Ab/Cry1Ac protein by the adult bees was also confirmed by ELISA measurement. In accordance with previous results obtained for Cry2A and Cry1C [31], the Cry protein concentration was lower after 10 d than after 5 d or 15 d. The reasons for this finding are not clear. It might, however, be linked to the fact that worker bee adults have the highest protein synthesis rates at an age of 8–16 days, which is caused by the high protein demand needed for the development of HPG which secretes royal jelly as food for queens and larvae [31,50].

In addition, it was confirmed that the bioactivity of the Cry1Ab/Cry1Ac protein in the *Bt* maize pollen used in the feeding bioassays did not change significantly during the two-day exposure period by a sensitive insect bioassay with *C. suppressalis* larvae. This finding is consistent with the results from earlier studies with *Bt* rice and maize pollen [31,35,36,37,38]. Since Cry1Ab/Cry1Ac was also detected in *P. japonica*, *C. nipponensis*, and *A. mellifera* after feeding *Bt* maize pollen for 21 d, 28 d, and 16 d, respectively, it is confirmed that all three test species had ingested bioactive Cry1Ab/Cry1Ac in our feeding bioassays. The results thus reinforce the conclusion that the three species are not sensitive to the recombinant Cry protein. Therefore, growing ZZM030 maize would pose a negligible risk to these non-target species.

## 4. Materials and Methods

### 4.1. Insects

Adults of *P. japonica* were collected at the experimental field station of the Institute of Plant Protection, Chinese Academy of Agricultural Sciences (CAAS), near Langfang City, Hebei Province, China (39.5°N, 116.7°E) in 2017, and subsequently maintained in the laboratory at 26 ± 1 °C, 75 ± 5% RH and a 16:8 h L:D photoperiod without introduction of field-collected insects for more than two generations. Both larvae and adults of *P. japonica* were reared on soybean seedlings infested with *Aphis glycines* (Matsumura) (Hemiptera: Aphididae). The aphids were renewed daily, ensuring enough food for the development of ladybirds. Newly hatched *P. japonica* larvae (<12 h after emergence) were used for the experiments. 

*C. nipponensis* were also collected at the experimental field station of the Institute of Plant Protection, CAAS, in 2017 and since maintained in the laboratory at 26 ± 1 °C, 75 ± 5% RH and a 16:8 h L:D photoperiod without introduction of field-collected insects. Larvae of *C. nipponensis* were fed on soybean seedlings infested with *A. glycines*. Adults were reared on an artificial diet containing sucrose and brewer’s yeast at a ratio of 1:1. The aphids were replaced daily, guaranteeing an ad libitum food supply for the larvae. Newly emerged (<24 h after emergence) *C. nipponensis* adults were used for the experiments. 

*A. mellifera* adults were obtained from a colony maintained at the Institute of Plant Protection, CAAS. After larvae matured in brood combs, they were placed in a climate chamber (30 ± 0.5 °C, 70 ± 1% RH, and no light). Newly emerged worker bees (<24 h after emergence) were used for the experiments.

Neonates of a *Bt*-susceptible strain of *Chilo suppressalis* (Walker) (Lepidoptera: Crambidae) were obtained from a colony that had been maintained on an artificial diet [51] for over 100 generations in our laboratory. 

### 4.2. Maize Plants and Pollen Collection

The *Bt* maize line ZZM030 and its corresponding non-transformed near isoline Xiang249 used for the experiments were developed by the China National Seed Group Company. ZZM030 plants express the Cry1Ab/Cry1Ac fusion protein under the control of the maize ubiquitin promotor. Cry1Ab/Cry1Ac is hybrid with domain I and domain II of Cry1Ab (UniProtKB: P0A370) and domain III of Cry1Ac (UniProtKB: P05068) optimized with corn genetic code (PCT/CN2016/090978) [52]. The maize lines were simultaneously planted in the China National Seed Group Company experiment field (Beijing, China). The plants were cultivated according to the common local agricultural practices but without insecticide sprays during the growing period. During the maize anthesis period, maize pollen was collected daily by shaking the maize tassels in a plastic bag. The pollen was air dried for 48 h at room temperature after being collected and subsequently sifted using a mesh size of 0.125 mm to remove anthers and contaminants. Pollen was stored at −80 °C until used.

### 4.3. Feeding Experiment with P. japonica

Experiments were conducted in climate chambers at 26 ± 1 °C, 75 ± 5% RH, and a 16:8 h L:D photoperiod. The test system for *P. japonica* was developed and validated in previous studies and has been successfully used to evaluate the potential effects of *Bt* rice and *Bt* maize pollen or purified Cry proteins on the predator [36,37,38,39]. The *P. japonica* larvae were individually confined in Petri dishes (6.0 cm diameter, 1.5 cm height). They were fed with pollen on the first day of each instar and then provided with a mixture of pollen and soybean aphids until development into the next instar. Adults were kept as single pairs in a Petri dish (6.0 cm diameter, 1.5 cm height) and fed with pollen or with a combination of pollen and soybean aphids every other day. The pollen was directly sprinkled on the bottom of the Petri dish, while the aphids were provided on 1 cm segments of heavily infested soybean seedlings. Pollen was replaced every 2 days and soybean aphids were replaced daily. In addition, an open 1.5 mL centrifuge tube containing solidified 1% agar solution was added to each dish as a water source. All food elements were provided ad libitum. For adults, folded paper tapes (1 cm width, 10 cm length) served as oviposition substrates. 

Seventy neonates of *P. japonica* were tested for each of the two treatments, i.e., pollen from Xiang249 maize (control) or from ZZM030 maize containing Cry1Ab/Cry1Ac. Larval survival rate, pupation rate, eclosion rate, and developmental time to pupa were recorded based on daily observations. When adults emerged, they were individually weighed using an electronic balance (CPA225D; Sartorius AG; readability = 0.1 mg, repeatability ± 0.1 mg). Subsequently, randomly selected pairs were continuously fed with *Bt* or control maize pollen and soybean aphids as described above. A total of 16–20 pairs of adult *P. japonica* were tested for each treatment. The survival, pre-oviposition period (days from emergence to the first oviposition), and total fecundity (number of eggs laid per female) were recorded and calculated based on daily observations. The experiment was terminated 21 days after the first oviposition occurred. Three samples (three adults per sample) were randomly selected and stored at −80 °C until used for ELISA analyses. 

### 4.4. Feeding Experiment with C. nipponensis

Experiments were conducted in a climatic chamber at 26 ± 1 °C, 75 ± 5% and a 16:8 h L:D photoperiod. According to previous studies [35,53], single pairs of *C. nipponensis* adults were confined in transparent plastic cylinders (8.0 cm diameter, 8.0 cm high). A layer of cotton gauze covered the mouth of the plastic cylinder to prevent the *C. nipponensis* adults from escaping and served as an oviposition substrate. A water-saturated cotton ball was placed on the bottom of each cylinder as a water source. Since mating of adult *C. nipponensis* generally occurs during the first few days after emergence, and re-mating is not required to ensure oviposition throughout the 28-day test period, males that died during the experiments were not replaced [53].

Freshly emerged adults of *C. nipponensis* from the same day were paired and kept in the containers as described above. Maize pollen and 2 M sucrose solution were added separately and ad libitum to the bottom of small Petri dishes (3.5 cm diameter, 1.2 cm high) and replaced every 2 days. Thirty pairs were tested for each of the two treatments, i.e., pollen from Xiang249 maize (control) or from ZZM030 maize containing Cry1Ab/Cry1Ac. The survival, pre-oviposition period, daily and total fecundity (number of eggs laid) were recorded and calculated based on daily observations. After 28 days, the feeding experiment was terminated. All of the remaining adults were collected and fresh weights were determined for each individual using an electronic balance (CPA224S, Sartorius, Germany; d = 0.1 mg, ± 0.1 mg). All insects were stored at −80 °C until they were used for confirming the presence of Cry1Ab/Cry1Ac protein in randomly selected insects by ELISA.

### 4.5. Feeding Experiment with A. mellifera

The test system used has been developed and validated previously [31,54]. Freshly emerged worker bees (<12 h after emergence) were confined in wooden cages (9 cm × 9 cm × 10 cm) which had wire netting on two sides for ventilation. The cages were kept in a climate chamber without light at 30 ± 1 °C, 75 ± 5% RH. The pollen-based diet in this experiment consisted of maize pollen mixed with honey at a ratio of 2:1 (w:w). The pollen-based diet was provided in 5 mL centrifuge tubes which were inserted into each cage. The sides of each tube had two holes that enabled the adult bees to access food. A second tube containing 60% sucrose solution was also inserted into each cage and featured six small holes that were formed with a needle to provide access to water.

Bees were provided with one of two treatments, i.e., pollen from Xiang249 maize (control) or from ZZM030 maize containing Cry1Ab/Cry1Ac. Thirty newly emerged worker bees were placed in each cage and each treatment was represented by four replicate cages. The pollen-based diet was replaced daily. Bee survival was recorded daily and the experiment was terminated after 16 days. 

In order to determine the potential effect of *Bt* maize pollen on hypopharyngeal gland development in *A. mellifera*, we also analyzed the acini diameter at day 10 following the methodology described previously [31,48,50]. Five bees were randomly collected from each cage (resulting in a total of 20 bees for each treatment) after 10 days for the measurement. The hypopharyngeal gland was dissected with forceps and placed in a 0.25 M sodium chloride solution isotonic to the hemolymph with the aid of a stereomicroscope (Olympus, SZX7, Tokyo; Japan). Ten acini randomly selected from each hypopharyngeal gland were measured per diameter, i.e., the short axis of the oval acinus, by a digital camera (MD50-T, Mshot, China) at 40× magnification [31].

For the ELISA measurement, 30 young bees were reared in one cage following the methods described above. After 5 d, 10 d, and 15 d of feeding, five bees per sampling time were collected randomly and frozen at −80 °C for later analysis.

### 4.6. Stability of Cry Protein in Maize Pollen during the Feeding Exposure

To determine the stability of the *Bt* protein in the maize pollen during the feeding exposure, three subsamples were collected of each (fresh pollen and pollen that was exposed to the conditions of the feeding experiments with *P. japonica* and *C. nipponensis* for 2 days). In addition, three subsamples were taken from the pollen diet immediately after it was prepared and after it had been exposed to *A. mellifera* for 1 day. The samples were stored at −80 °C until further use.

### 4.7. ELISA Measurements

For all experiments, the concentrations of Cry1Ab/Cry1Ac in maize pollen and in insect samples were measured by ELISA using Cry1Ab/Cry1Ac detection kits (QuantiPlate^TM^ Kit, Catalog No. AP 007) from EnviroLogix Inc. (Portland, ME, USA). Before the analysis, insect samples were washed in phosphate buffered saline Tween-20 (PBST) (provided with the kit) to remove *Bt* maize pollen from their surface. For Cry protein extraction, samples of insects or maize pollen were weighed and mixed with PBST at a ratio of 1:10 to 1:100 (mg of sample:mL of buffer) in 1.5 mL centrifuge tubes. The samples were then fully ground by hand on ice using an electric grinding rod. After centrifugation and appropriate dilution of the supernatants, the ELISA was performed according to the manufacturer’s instruction. The optical density (OD) values were read by a microplate spectrophotometer (PowerWave XS2, BioTek, Winooski, VT USA). The concentrations of Cry1Ab/Cry1Ac were calculated by calibrating the OD values to a range of Cry1Ab/Cry1Ac standards (0 ppm, 0.13 ppm, 0.25 ppm, 0.50 ppm and 1.00 ppm) made from a standard solution provided with the kit.

### 4.8. Bioactivity of Cry1Ab/Cry1Ac Protein in Bt Maize Pollen

The bioactivity of the Cry1Ab/Cry1Ac protein in *Bt* maize pollen before and after exposure in a climate chamber at 26 ± 1 °C, 75 ± 5% RH and a 16:8 h L:D photoperiod for 2 days was determined in a sensitive insect bioassay using larvae of a *Bt*-susceptible *C. suppressalis* strain. Three hundred milligrams of pollen from Xiang249 (control) or ZZM030 was thoroughly incorporated into 5.7 g of artificial diet for *C. suppressalis* larvae. A slice of this diet was placed in a Petri dish (9 cm diameter, 1 cm height) and 10 neonates of *C. suppressalis* (<12 h after emergence) were added. Subsequently, the Petri dishes were sealed with parafilm and reinforced with surgical tape. After 7 days, the *C. suppressalis* larvae were weighed (10 individuals were pooled and weighed as one sample) on a microbalance. Three Petri dishes (replicates) were tested for each treatment.

### 4.9. Data Analysis

Pair-wise statistical comparisons were made between the two pollen treatments in all experiments. Chi-square tests were used to compare *P. japonica* pupation rates and eclosion rates and *C. nipponensis* adult survival (females and males separately). Mann–Whitney U-tests were used to compare *P. japonica* larval developmental times and *P. japonica* pre-oviposition periods because such data did not satisfy the assumptions for parametric analyses (normal distribution of residuals and homogeneity of error variances). Bee survival rates were compared by Kaplan–Meier analysis for abnormal distribution of the data. The mean acini diameters of hypopharyngeal glands were compared by Student’s *t*-test.

Data on *P. japonica* and *C. nipponensis* adult fresh weight and total fecundity were compared using Student’s *t*-test. In addition, Student’s *t*-tests were conducted to compare the weights of *C. suppressalis* larvae that were fed with artificial diets containing different pollen treatments.

All statistical analyses were conducted using the software package SPSS (version 19; SPSS, Inc., Chicago, IL, USA).

## Figures and Tables

**Table 1:** Life-table parameters of *Propylea japonica* larvae fed pollen from *Bt* (ZZM030) or the corresponding non-*Bt* (Xiang249) maize. Number of replicates is given in parentheses.

Parameters	Non-*Bt* Pollen	*Bt* Pollen	Statistics
Larval survival rate (%) ^a^	91.43 (70)	92.86 (70)	*X*^2^ = 0.10, *P* = 0.75
Pupation rate (%) ^a^	75.71 (70)	90.00 (70)	*X*^2^ = 5.03, *P* = 0.03
Eclosion rate (%) ^a^	90.57 (53)	95.24 (63)	*X*^2^ = 0.98, *P* = 0.32
Days to pupa (days ± SE) ^b^	9.34 ± 0.20 (52)	9.07 ± 0.14 (63)	*U* = 1485, *P* = 0.37
Female fresh weight (mg ± SE) ^c^	6.07 ± 0.22 (23)	6.26 ± 0.17 (34)	*t* = −0.68, *P* = 0.50
Male fresh weight (mg ± SE) ^c^	4.60 ± 0.16 (25)	5.21 ± 0.15 (26)	*t* = −2.74, *P* = 0.01
Pre-oviposition period (days ± SE) ^b^	4.81 ± 0.61 (16)	4.65 ± 0.42 (20)	*U* = 145, *P* = 0.39
Total fecundity per pair over 21 d (eggs ± SE) ^c^	120.81 ± 10.68 (16)	129.00 ± 9.23 (20)	*t* = −0.71, *P* = 0.48

^a^ χ^2^ test. ^b^ Mann–Whitney U-test. ^c^ Student’s *t*-test.

**Table 2:** Life-table parameters of *Chrysoperla nipponensis* adults fed pollen from *Bt* (ZZM030) or the corresponding non-*Bt* (Xiang249) maize over a period of 28 days. Number of replicates is given in parentheses.

Parameters	*Bt* Pollen	Non-*Bt* Pollen	Statistics
Male survival (%) ^a^	90 (30)	90 (30)	*X*^2^ = 0.48, *P* = 0.49
Female survival (%) ^a^	79.31 (29)	71.43 (28)	*X*^2^ = 0.48, *P* = 0.49
Pre-oviposition period (days ± SE) ^b^	10.19 ± 0.55 (26)	9.26 ± 0.56 (27)	*U* = 289, *P* = 0.26
Total fecundity (eggs ± SE) ^c^	63.96 ± 7.56 (25)	79.04 ± 10.25 (25)	*t* = −1.18, *P* = 0.24
Male fresh weight (mg ± SE) ^c^	4.61 ± 0.18 (27)	4.59 ± 0.15 (27)	*t* = 0.08, *P* = 0.93
Female fresh weight (mg ± SE) ^c^	6.94 ± 0.32 (23)	6.87 ± 0.26 (20)	*t* = 0.16, *P* = 0.87

^a^ Chi-square test. ^b^ Mann–Whitney U-test.^. c^ Student’s *t*-test.

## References

[1] Hellmich R.L., Albajes R., Bergvinson D., Prasifka J.R., Wang Z.Y., Weiss M.J., Romeis J., Kennedy G.G., Shelton A.M. (2008). The present and future role of insect-resistant genetically modified maize in IPM. Integration of Insect-Resistant Genetically Modified Crops within IPM Programs.

[2] ISAAA (2017). Global Status of Commercialized Biotech/GM Crops in 2017: Biotech Crop Adoption Surges as Economic Benefits Accumulate in 22 Years.

[3] Dively G.P., Venugopal P.D., Bean D., Whalen J., Holmstrom K., Kuhar T.P., Doughty H.B., Patton T., Cissel W., Hutchison W.D. (2018). Regional pest suppression associated with widespread *Bt* maize adoption benefits vegetable growers. Proc. Natl. Acad. Sci. USA.

[4] Hutchison W.D., Burkness E.C., Mitchell P.D., Moon R.D., Leslie T.W., Fleischer S.J., Abrahamson M., Hamilton K.L., Steffey K.L., Gray M.E. (2010). Areawide suppression of European corn borer with *Bt* maize reaps savings to non-*Bt* maize growers. Science.

[5] Garcia-Alonso M., Jacobs E., Raybould A., Nickson T.E., Sowig P., Willekens H., Van der Kouwe P., Layton R., Amijee F., Fuentes A.M. (2007). A tiered system for assessing the risk of genetically modified plants to non-target organisms. Environ. Biosaf. Res..

[6] Li Y., Peng Y., Hallerman E.M., Wu K. (2014). Biosafety management and commercial use of genetically modified crops in China. Plant Cell Rep..

[7] Romeis J., Bartsch D., Bigler F., Candolfi M.P., Gielkens M.M.C., Hartley S.E., Hellmich R.L., Huesing J.E., Jepson P.C., Layton R. (2008). Assessment of risk of insect-resistant transgenic crops to nontarget arthropods. Nat. Biotechnol..

[8] Sanvido O., Romeis J., Gathmann A., Gielkens M., Raybould A., Bigler F. (2012). Evaluating environmental risks of genetically modified crops: Ecological harm criteria for regulatory decision-making. Environ. Sci. Policy.

[9] Duan J.J., Marvier M., Huesing J., Dively G., Huang Z.Y. (2008). A meta-analysis of effects of *Bt* crops on honey bees (Hymenoptera: Apidae). PLoS ONE.

[10] Icoz I., Stotzky G. (2008). Fate and effects of insect-resistant *Bt* crops in soil ecosystems. Soil Biol. Biochem..

[11] Li Y.H., Romeis J., Wu K.M., Peng Y.F. (2014). Tier-1 assays for assessing the toxicity of insecticidal proteins produced by genetically engineered plants to non-target arthropods. Insect Sci..

[12] Marvier M., Mccreedy C., Regetz J., Kareiva P. (2007). A meta-analysis of effects of *Bt* cotton and maize on nontarget invertebrates. Science.

[13] Naranjo S.E. (2009). Impacts of *Bt* crops on non-target invertebrates and insecticide use patterns. CAB Rev. Perspect. Agric. Vet. Sci. Nutr. Nat. Resour..

[14] Romeis J., Naranjo S.E., Meissle M., Shelton A.M. (2018). Genetically engineered crops help support conservation biological control. Biol. Contr..

[15] Romeis J., Meissle M., Bigler F. (2006). Transgenic crops expressing *Bacillus thuringiensis* toxins and biological control. Nat. Biotechnol..

[16] Wolfenbarger L.L., Naranjo S.E., Lundgren J.G., Bitzer R.J., Watrud L.S. (2008). *Bt* crop effects on functional guilds of non-target arthropods: A meta-analysis. PLoS ONE.

[17] Wang Y.B., Lang Z.H., Zhang J., He K.L., Song F.P., Huang D.F. (2008). Ubi1 intron-mediated enhancement of the expression of *Bt cry1Ah* gene in transgenic maize (*Zea mays* L.). Chin. Sci. Bull..

[18] Lv X., Wang H., Zeng X., Yang X., Weng J., Di H., Guo Y., Wang Z., Li X. (2013). Research and application of transgenic *Bt* corn for insect resistance. Crops.

[19] Devos Y., Romeis J., Luttik R., Maggiore A., Perry J.N., Schoonjans R., Streissl F., Tarazona J.V., Brock T.C. (2015). Optimising environmental risk assessments: Accounting for ecosystem services helps to translate broad policy protection goals into specific operational ones for environmental risk assessments. EMBO Rep..

[20] Romeis J., Raybould A., Bigler F., Candolfi M.P., Hellmich R.L., Huesing J.E., Shelton A.M. (2013). Deriving criteria to select arthropod species for laboratory tests to assess the ecological risks from cultivating arthropod-resistant genetically engineered crops. Chemosphere.

[21] Romeis J., Meissle M., Álvarez-Alfageme F., Bigler F., Bohan D.A., Devos Y., Malone L.A., Pons X., Rauschen S. (2014). Potential use of an arthropod database to support the non-target risk assessment and monitoring of transgenic plants. Transgenic Res..

[22] Bai Y.Y., Jiang M.X., Cheng J.A. (2005). Effects of transgenic *cry1Ab* rice pollen on fitness of *Propylea japonica* (Thunberg). J. Pest Sci..

[23] Zhou K.J., Xiang J.B. (1987). Observations on the efficacy of spiders and ladybirds against aphids in the seedling stage of cotton in the cotton fields. Nat. Enemies Insects.

[24] Zhang S.Y., Li D.M., Cui J., Xie B.Y. (2006). Effects of *Bt*-toxin Cry1Ac on *Propylaea japonica* Thunberg (Col., Coccinellidae) by feeding on *Bt*-treated *Bt*-resistant *Helicoverpa armigera* (Hubner) (Lep., Noctuidae) larvae. J. Appl. Entomol..

[25] Li Y.H., Zhang Q.L., Liu Q.S., Meissle M., Yang Y., Wang Y.N., Hua H.X., Chen X.P., Peng Y.F., Romeis J. (2017). *Bt* rice in China—Focusing the nontarget risk assessment. Plant Biotechnol. J..

[26] Li Y., Meissle M., Romeis J. (2010). Use of maize pollen by adult *Chrysoperla carnea* (Neuroptera: Chrysopidae) and fate of Cry proteins in *Bt* -transgenic varieties. J. Insect Physiol..

[27] Romeis J., Meissle M., Naranjo S., Li Y., Bigler F. (2014). The end of a myth—*Bt* (Cry1Ab) maize does not harm green lacewings. Front. Plant Sci..

[28] Han P., Niu C.Y., Biondi A., Desneux N. (2012). Does transgenic Cry1Ac+CpTI cotton pollen affect hypopharyngeal gland development and midgut proteolytic enzyme activity in the honey bee *Apis mellifera* L. (Hymenoptera, Apidae)?. Ecotoxicology.

[29] Hendriksma H.P., Kuting M., Hartel S., Nather A., Dohrmann A.B., Steffan-Dewenter I., Tebbe C.C. (2013). Effect of stacked insecticidal Cry proteins from maize pollen on nurse bees (*Apis mellifera carnica*) and their gut bacteria. PLoS ONE.

[30] Rose R., Dively G.P., Pettis J. (2007). Effects of *Bt* corn pollen on honey bees: Emphasis on protocol development. Apidologie.

[31] Wang Y., Dai P., Chen X., Romeis J., Shi J., Peng Y., Li Y. (2016). Ingestion of *Bt* rice pollen does not reduce the survival or hypopharyngeal gland development of *Apis mellifera* adults. Environ. Toxicol. Chem..

[32] Li Y., Meissle M., Romeis J. (2008). Consumption of *Bt* maize pollen expressing Cry1Ab or Cry3Bb1 does not harm adult green Lacewings, *Chrysoperla carnea* (Neuroptera: Chrysopidae). PLoS ONE.

[33] Malone L.A. (2004). Potential effects of GM crops on honey bee health. Bee World.

[34] Romeis J., Hellmich R.L., Candolfi M.P., Carstens K., De Schrijver A., Gatehouse A.M.R., Herman R.A., Huesing J.E., Mclean M.A., Raybould A. (2011). Recommendations for the design of laboratory studies on non-target arthropods for risk assessment of genetically engineered plants. Transgenic Res..

[35] Wang Y., Li Y., Romeis J., Chen X., Zhang J., Chen H., Peng Y. (2012). Consumption of *Bt* rice pollen expressing Cry2Aa does not cause adverse effects on adult *Chrysoperla sinica* Tjeder (Neuroptera: Chrysopidae). Biol. Contr..

[36] Li Y., Zhang X., Chen X., Romeis J., Yin X., Peng Y. (2015). Consumption of *Bt* rice pollen containing Cry1C or Cry2A does not pose a risk to *Propylea japonica* (Thunberg) (Coleoptera: Coccinellidae). Sci. Rep..

[37] Li Y., Liu Y., Yin X., Romeis J., Song X., Chen X., Geng L., Peng Y., Li Y. (2017). Consumption of *Bt* maize pollen containing Cry1Ie does not negatively affect *Propylea japonica* (Thunberg) (Coleoptera: Coccinellidae). Toxins.

[38] Liu Y., Liu Q., Wang Y., Chen X., Song X., Romeis J., Li Y., Peng Y. (2016). Ingestion of *Bt* corn pollen containing Cry1Ab/2Aj or Cry1Ac does not harm *Propylea japonica* larvae. Sci. Rep..

[39] Zhang X.J., Li Y.H., Romeis J., Yin X.M., Wu K.M., Peng Y.F. (2014). Use of a pollen-based diet to expose the ladybird beetle *Propylea japonica* to insecticidal proteins. PLoS ONE.

[40] Meissle M., Zünd J., Waldburger M., Romeis J. (2014). Development of *Chrysoperla carnea* (Stephens) (Neuroptera: Chrysopidae) on pollen from *Bt*-transgenic and conventional maize. Sci. Rep..

[41] Lang A., Vojtech E. (2006). The effects of pollen consumption of transgenic *Bt* maize on the common swallowtail, *Papilio machaon* L. (Lepidoptera, Papilionidae). Basic Appl. Ecol..

[42] Li Y., Wang Y., Romeis J., Liu Q., Lin K., Chen X., Peng Y. (2013). *Bt* rice expressing Cry2Aa does not cause direct detrimental effects on larvae of *Chrysoperla sinica*. Ecotoxicology.

[43] Li Y., Chen X., Hu L., Romeis J., Peng Y. (2014). *Bt* rice producing Cry1C protein does not have direct detrimental effects on the green lacewing *Chrysoperla sinica* (Tjeder). Environ. Toxicol. Chem..

[44] Tian J.C., Wang X.P., Long L.P., Romeis J., Naranjo S.E., Hellmich R.L., Wang P., Earle E.D., Shelton A.M. (2013). *Bt* crops producing Cry1Ac, Cry2Ab and Cry1F do not harm the green lacewing, *Chrysoperla rufilabris*. PLoS ONE.

[45] Van den Berg J., Warren J.F., du Plessis H. (2017). The potential effect of *Bt* maize on *Chrysoperla pudica* (Neuroptera: Chrysopidae). Environ. Entomol..

[46] Hilbeck A., Moar W.J., Pusztai-Carey M., Filippin A., Bigler F. (1998). Toxicity of *Bacillus thuringiensis* Cry1Ab toxin to the predator *Chrysoperla carnea* (Neuroptera: Chrysopidae). Environ. Entomol..

[47] Mason C.E., Sheldon J.K., Pesekadke G., Slabaugh B. (2008). Assessment of Chrysoperla plorabunda longevity, fecundity, and egg viability when adults are fed transgenic *Bt* corn pollen. J. Agric. Urban Entomol..

[48] Babendreier D., Kalberer N.M., Romeis J., Fluri P., Mulligan E., Bigler F. (2005). Influence of *Bt*-transgenic pollen, *Bt*-toxin and protease inhibitor (SBTI) ingestion on development of the hypopharyngeal glands in honeybees. Apidologie.

[49] Malone L.A., Todd J.H., Burgess E.P.J., Christeller J.T. (2004). Development of hypopharyngeal glands in adult honey bees fed with a *Bt*-toxin, a biotin-binding protein and a protease inhibitor. Apidologie.

[50] Sagili R.R., Pankiw T., Zhusalzman K. (2005). Effects of soybean trypsin inhibitor on hypopharyngeal gland protein content, total midgut protease activity and survival of the honey bee (*Apis mellifera* L.). J. Insect Physiol..

[51] Han L.Z., Li S.B., Peng Y.F., Hou M.L. (2012). New artificial diet for continuous rearing of *Chilo suppressalis*. Ann. Entomol. Soc. Am..

[52] Liu B.L., Tan C., Yang Q.Q., Xu J.T., Wen Q., Qiu L., Ma C.L., Zhang W.G. (2016). Gene for Encoding *Bacillus thuringiensis* Crystal Proteins and Application Thereof.

[53] Xu Y.J. (2001). Diapause Mechanism and Application of *Chrysoperla sinica* (Tjeder). Ph.D. Dissertation.

[54] Niu L., Ma Y., Mannakkara A., Zhao Y., Ma W., Lei C., Chen L. (2013). Impact of single and stacked insect-resistant *Bt*-cotton on the honey bee and silkworm. PLoS ONE.

